# Gas-phase synthesis of benzene via the propargyl radical self-reaction

**DOI:** 10.1126/sciadv.abf0360

**Published:** 2021-05-21

**Authors:** Long Zhao, Wenchao Lu, Musahid Ahmed, Marsel V. Zagidullin, Valeriy N. Azyazov, Alexander N. Morozov, Alexander M. Mebel, Ralf I. Kaiser

**Affiliations:** 1 Department of Chemistry, University of Hawaii at Manoa, Honolulu, HI 96822, USA.; 2 Chemical Sciences Division, Lawrence Berkeley National Laboratory, Berkeley, CA 94720, USA.; 3 Lebedev Physical Institute, Samara 443011, Russian Federation.; 4 Samara National Research University, Samara 443086, Russian Federation.; 5 Department of Chemistry and Biochemistry, Florida International University, Miami, FL 33199, USA.

## Abstract

Polycyclic aromatic hydrocarbons (PAHs) have been invoked in fundamental molecular mass growth processes in our galaxy. We provide compelling evidence of the formation of the very first ringed aromatic and building block of PAHs—benzene—via the self-recombination of two resonantly stabilized propargyl (C_3_H_3_) radicals in dilute environments using isomer-selective synchrotron-based mass spectrometry coupled to theoretical calculations. Along with benzene, three other structural isomers (1,5-hexadiyne, fulvene, and 2-ethynyl-1,3-butadiene) and *o*-benzyne are detected, and their branching ratios are quantified experimentally and verified with the aid of computational fluid dynamics and kinetic simulations. These results uncover molecular growth pathways not only in interstellar, circumstellar, and solar systems environments but also in combustion systems, which help us gain a better understanding of the hydrocarbon chemistry of our universe.

## Teaser

The propargyl self-reaction leading to C_6_H_6_ isomers is critical in our understanding of the evolution of carbon in our galaxy.

## INTRODUCTION

**Fig. 1:**
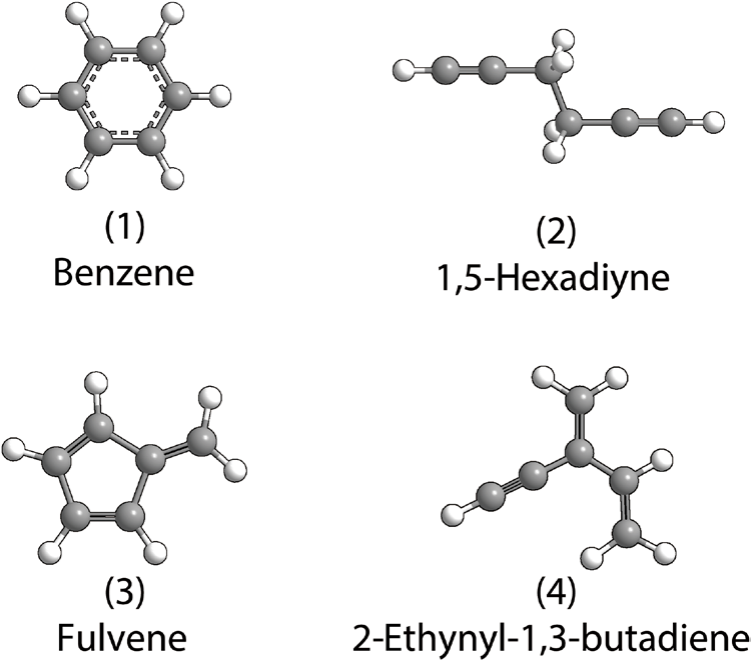
Molecular structures of the benzene molecule along with key structural isomers.

The benzene molecule [C_6_H_6_ **(1)**] isolated 175 years ago by Hofmann (*1*) has been recognized as the fundamental molecular building block of polycyclic aromatic hydrocarbons (PAHs)—organic molecules composed of multiple fused benzenoid rings (Fig. 1) (*2*). PAHs, along with their protonated (*3*), ionized (*4*), alkylated (*5*), and (de)hydrogenated (*6*) counterparts, are ubiquitous in the universe and have been associated with the unidentified infrared emission bands (*7*–*9*) and with the diffuse interstellar bands (DIBs)—discrete absorption features superimposed on the interstellar extinction curve (*9*, *10*). These aromatics, identified in carbonaceous chondrites such as Murchison, Allende, and Orgueil as products of high-temperature (few 1000 K) synthesis in carbon-rich asymptotic giant branch stars, could represent the missing link between resonantly stabilized free radicals (RSFRs) such as propargyl (C_3_H_3_^•^) and carbonaceous nanoparticles (interstellar grains) (*11*) incorporating up to 30% of the cosmic carbon budget (*9*, *12*–*15*). Whereas these PAHs have been conjectured to be possible starting materials for an abiotic synthesis of biorelevant material vital to the earliest forms of life (*16*–*19*), on present-day Earth, PAHs along with carbonaceous nanoparticle (soot) as their descendants represent undesirable toxic, often carcinogenic by-products released in incomplete combustion (*20*). However, there is a critical lack of a fundamental understanding of the high-temperature formation mechanisms of the aromatic benzene molecule [C_6_H_6_ **(1)**], the simplest building block of all PAHs.

The recombination of two propargyl radicals leading to benzene formation has been predicted theoretically (Supplementary Materials) (*21*–*31*); however, there is no experimental verification of the self-recombination of RSFRs and the direct in situ detection of benzene along with its isomers limited to the very first aromatic ring (Supplementary Materials; table S1). Given the possible number of isomers present at the benzene mass [mass/charge ratio (*m/z*) = 78] and its importance in astrophysics, combustion, and the environment, experimental studies are clearly required, which allow the study of the initial steps in the propargyl radical self-reaction leading to the very first ring, while simultaneously excluding molecular mass growth processes to aromatics beyond benzene. This approach provides an experimental benchmark for the conversion of propargyl radicals to benzene along with its structural isomers and for the self-recombination of RSFRs in general.

Here, we provide compelling evidence of the very first in situ detection of the benzene molecule [C_6_H_6_ **(1)**] along with three structural isomers [1,5-hexadiyne **(2)**, fulvene **(3)**, and 2-ethynyl-1,3-butadiene **(4)**] (Fig. 1) formed via the barrierless self-recombination of the propargyl radical (C_3_H_3_^•^) (reaction 1) at conditions mimicking combustion and circumstellar environments using a high-temperature chemical reactor coupled with isomer-specific detection using tunable vacuum ultraviolet (VUV) light. Computational fluid dynamics (CFD) and kinetic simulations of the reactor reveal a second channel (reaction 2) leading to the phenyl radical (C_6_H_5_^•^), which subsequently reacts to the *o*-benzyne molecule (C_6_H_4_) (reaction 3). The self-recombination of two propargyl radicals, important in circumstellar envelopes (*32*, *33*) and combustion environments (*34*), provides a template of a reaction class for aromatic molecules with a benzene core to form from acyclic RSFR precursors. The key products benzene (C_6_H_6_) (*35*), the phenyl radical (C_6_H_5_^•^) (*36*), and *o*-benzyne (C_6_H_4_) (*37*) participate in essential mass growth processes from the very first aromatic ring to complex PAHs, thus providing a better understanding of the chemistry of carbon in the universe

$$C_{3}{H_{3}}^{•}+C_{3}{H_{3}}^{•}\to C_{6}H_{6}$$ (1)

$$C_{3}{H_{3}}^{•}+C_{3}{H_{3}}^{•}\to C_{6}{H_{5}}^{•}+H$$ (2)

$$C_{6}{H_{5}}^{•}+H^{•}\to o‐C_{6}H_{4}+H_{2}$$ (3a)

$$C_{6}{H_{5}}^{•}\to C_{6}H_{4}+H^{•}$$ (3b)

## RESULTS AND DISCUSSION

### Mass Spectra

**Fig. 2:**
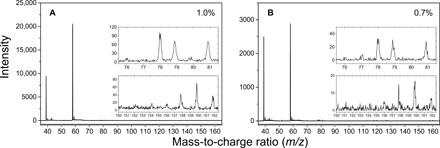
Photoionization mass spectra for the propargyl recombination reaction recorded at the photon energy of 10.5 eV. The conditions for the experiments are as follows: (**A**) The initial mole fraction of propargyl bromide is 1.0%, the total inlet pressure is 200 torr, and the reactor temperature is 1343 ± 10 K. (**B**) The initial mole fraction of propargyl bromide is 0.7%, the total inlet pressure is 300 torr, and the reactor temperature is 1273 ± 10 K.

The propargyl radical self-reaction was explored in a high-temperature chemical reactor (*38*–*40*). This reactor consists of a heated silicon carbide (SiC) tube and is incorporated within the source chamber of a molecular beam apparatus equipped with a Wiley-McLaren reflectron time-of-flight mass spectrometer (Re-TOF-MS). Thermally labile propargyl bromide (C_3_H_3_Br) precursor molecules were pyrolyzed quantitatively in situ via cleavage of a weak carbon-bromine bond to generate helium-seeded propargyl radicals at temperatures of 1273 and 1343 K (Supplementary Materials; table S2). In this reactor, propargyl bromide dissociates to the propargyl radical plus atomic bromine. This approach to exploit thermally labile precursors is ideally suited to selectively prepare hydrocarbon radicals via carbon-bromine bond cleavages (*41*, *42*). Inlet pressures into the reactor were varied at 200 and 300 torr to generate propargyl radical fractions with propargyl radical precursors as low as 0.7% at the inlet of the reactor. Mass spectra, providing information of the respective molecular formulas of the reaction products, collected at a photoionization energy of 10.50 eV are displayed in Fig. 2, while the full mass spectra of all propargyl radical recombination mass spectra are reported in fig. S1. Reference (“blank”) experiments of helium-seeded propargyl bromide without heating the SiC tube confirming that the products are not contaminations from the reactants are reported in the Supplementary Materials (fig. S2). The mass spectra in Fig. 2 reveal ion counts at *m/z* = 39, 40, 76, 77, 78, 79, and 81. No signal is observed at *m/z* = 118/120 (C_3_H_3_^79^Br/C_3_H_3_^81^Br), demonstrating that propargyl bromide decomposed quantitatively to propargyl (*m/z* = 39) plus atomic bromine (*m/z* = 79 and 81) (Fig. 2). The aforementioned ions can be connected to species with the chemical formulas of C_3_H_3_ [39 atomic mass units (amu)], ^13^CC_2_H_3_/C_3_H_4_ (40 amu), C_6_H_4_ (76 amu), ^13^CC_5_H_4_ /C_6_H_5_ (77 amu), C_6_H_6_ (78 amu), ^13^CC_5_H_6_/^79^Br (79 amu), and ^81^Br (81 amu) in each system. With the exception of signal at *m/z* = 39 arising from photo fragmentation of the propargyl bromide precursor, these ion counts are absent in the control experiments, suggesting that the ions detected can be connected to actual reaction products (fig. S2). Note that the dilute propargyl radical reactants at 0.7 and 1.0% reveal a critical prerequisite to limit hydrocarbon molecular mass growth processes to *m/z* = 78 (C_6_H_6_) (Fig. 2 and fig. S1, A and B). Higher concentrations of the precursor concentrations of up to 4.8% result in molecular mass growth mechanisms beyond *m/z* = 78 as evident from ion counts observed at *m/z* = 102, 116, and 152 (fig. S1). It should be noticed that the ion counts at *m/z* = 102, 116, and 152 were only observed when the propargyl bromide reservoir was cooled down at 233 K to generate precursor concentrations of 3.2 and 4.8%. The signal at *m/z* = 156, 158, 160, and 162 are attributed to phenyl bromide (C_6_H_5_^79^Br, 156 amu; C_6_H_5_^81^Br, 158 amu) and molecular bromine (^79^Br_2_, 158 amu; ^79^Br^81^Br, 160 amu; ^81^Br_2_, 162 amu), which come from the recombination reactions of atomic bromine, the most abundant initial radical from the cleavage of precursor propargyl bromide. The mass spectra changes upon precursor concentration reveal that dilute concentrations of radicals are required to eliminate molecular mass growth processes beyond benzene.

### Photoionization efficiency curves

**Fig. 3:**
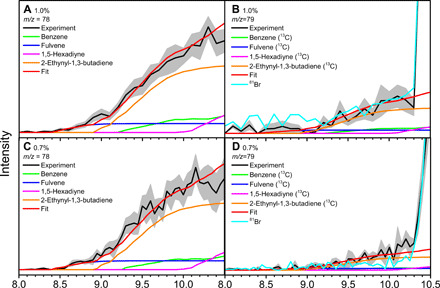
Photoionization efficiency (PIE) curves for signal at *m/z* = 78 and 79 in the propargyl recombination system. At the experiment conditions, the initial mole fraction of propargyl bromide is 1.0% (**A** and **B**) and 0.7% (**C** and **D**), respectively. Black: experimentally derived PIE curves; colored lines (green, blue, purple, and orange): reference PIE curves; and red lines: overall fit. The light blue line is the PIE curve for *m/z* = 81 observed in this work. The overall error shades consist of two parts: ±10% based on the accuracy of the photodiode and a 1 σ error of the PIE curve averaged over the individual scans.

To identify the structural isomer(s) formed in the propargyl self-recombination, we use photoionization efficiency (PIE) curves, which report the intensity of a well-defined ion of a specific *m/z* ratio as a function of photon energy (Fig. 3 and figs. S3 to S8), which then can be fit with a (linear combination of) reference curve(s) of distinct isomer(s). First, the PIE curves at *m/z* = 78 for precursor concentrations of 0.7 and 1.0% (Fig. 3, A and C, and fig. S7) can be fit well with a linear combination of the reference curves of four C_6_H_6_ isomers: benzene **(1)**, 1,5-hexadiyne **(2)**, fulvene **(3)**, and 2-ethynyl-1,3-butadiene **(4)**. The onset of ion counts at 8.40 ± 0.05 eV agrees closely with the adiabatic ionization energy (AIE) of the fulvene isomer of 8.36 ± 0.02 eV (*41*). The contribution of fulvene is critical to account for the ion counts at *m/z* = 78 collected at photon energies up to 8.9 eV. 2-Ethynyl-1,3-butadiene **(4)**, benzene **(1)**, and 1,5-hexadiyne **(2)** have discrete onsets at 8.95 ± 0.05 eV, 9.25 ± 0.05 eV, and 9.95 ± 0.05 eV as matched by their recorded AIE of 8.95 eV (*43*), 9.24 ± 0.05 eV (*44*), and 9.98 ± 0.05 eV (*45*), respectively. Second, the PIE curves of *m/z* = 79 (Fig. 3, B and D, and fig. S8) are quite distinct from those at *m/z* = 78. The PIE at *m/z* = 79 requires a linear combination of the four C_6_H_6_ isomers **(1)** to **(4)** arising from the natural ^13^C abundance (^13^CC_5_H_6_) along with ion counts from single ionized bromine-79 (^79^Br^+^). This is evident from the overlaid PIE curves of ^81^Br^+^, which nicely matches the signal at *m/z* = 79 at photon energies from about 10.3 to 10.5 eV. Third, the PIE curves of *m/z* = 76 (fig. S5) and 77 (fig. S6) can be replicated with the reference curve (*46*) of the *o*-benzyne isomer (C_6_H_4_, 76 amu; ^13^CC_5_H_4_, 77 amu); the onset at 9.00 ± 0.05 eV correlates with the AIE of *o*-benzyne of 9.03 eV (*47*). Fourth, the PIE curves of *m/z* = 39 (fig. S3) match the reference PIE curves of the propargyl radical exceptionally well. The onset of the ion counts of 8.65 ± 0.05 eV agrees with the AIE of propargyl recorded to be of 8.70 ± 0.02 eV (*48*). Last, the PIEs at *m/z* = 40 (fig. S4) could be replicated by a linear combination of three reference curves. Ion counts in the range of 8.3 to 9.9 eV could be fit nicely with the PIE curve of ^13^C-propargyl (^13^CC_2_H_3_); additional ion counts from the PIE of allene (H_2_CCCH_2_) were necessary to replicate the range from 9.8 to 10.3 eV, whereas contributions from a second C_3_H_4_ isomer—methylacetylene (CH_3_CCH)—were critical to replicate the overall PIE at *m/z* = 40 in the range of 10.3 to 10.5 eV. To conclude, the detailed analysis of the PIE curves resulted in the identification of distinct molecules [benzene **(1)**, 1,5-hexadiyne **(2)**, fulvene **(3)**, 2-ethynyl-1,3-butadiene **(4)**, C_6_H_6_; *o*-benzyne, C_6_H_4_ **(8)**] along with atomic hydrogen abstraction/recombination products of the propargyl radical, i.e., methylacetylene (CH_3_CCH) and allene (H_2_CCCH_2_), in those systems with propargyl radical reactant concentrations of 0.7 and 1.0% (Supplementary Materials; table S2). PIE curves of higher *m/z* ratios (*m/z* = 102, 116, 152, 156, 158, 160, and 162) are provided in the Supplementary Materials (figs. S9 to S15).

### CFD and kinetic simulations

**Fig. 4:**
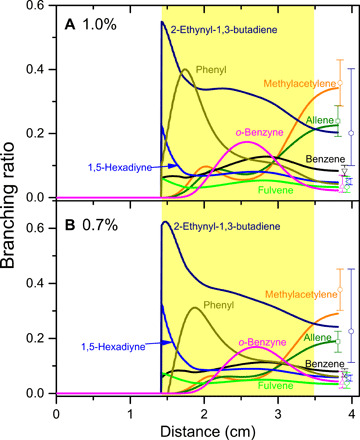
Simulated profiles (lines) and experimental measurements (open symbols with the error bars) of the branching ratios of benzene and its isomers together with the methylacetylene, allene, phenyl, and *o*-benzyne along the reactor axis. The experimental measurements are presented at different *x* positions for clarity. The simulation was performed for the experiment condition with (**A**) an initial mole fraction of propargyl bromide of 1.0%, an inlet pressure of 200 torr, a reactor temperature of 1343 K and (**B**) an initial mole fraction of propargyl bromide of 0.7%, an inlet pressure of 300 torr, and a reactor temperature at 1273 K. The yellow shaded area defines the heated section of the SiC tube.

**Fig. 5:**
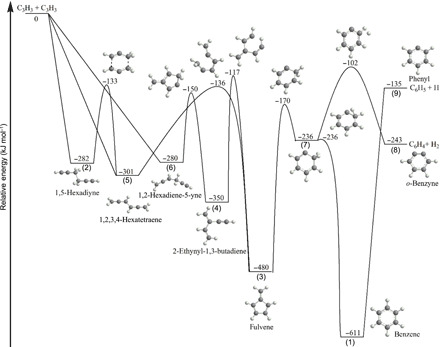
Simplified potential energy surface for the propargyl radical self-reaction modified from (*31*). Relative energies with respect to the initial reactants are given in kilojoules per mol.

CFD simulations of the “pyrolytic reactor” are performed to explain the formation of the molecules identified previously with mass spectrometry [benzene **(1**), 1,5-hexadiyne **(2)**, fulvene **(3)**, 2-ethynyl-1,3-butadiene **(4)**, C_6_H_6_; *o*-benzyne, C_6_H_4_ **(8)**] along with methylacetylene (CH_3_CCH) and allene (H_2_CCCH_2_). These simulations model the temperature and pressure profiles within the reactor and also incorporate a kinetic reaction model to rationalize the experimentally derived branching ratios of the reaction products (Fig. 4 and the Supplementary Materials; fig. S16 and tables S3 and S4) (*49*). The validity and success of this approach have been demonstrated previously by benchmarking the formation of naphthalene (C_10_H_8_) and [4]-helicene (C_18_H_12_) (*49*–*51*). The simulations are restricted to those systems with propargyl radical concentrations at 0.7 and 1.0% (table S2) since our main interest is to understand the fundamental reaction mechanisms leading to the formation of the very first aromatic building block of PAHs—benzene—and possibly to the phenyl radical along with *o*-benzyne [reactions (1) to (3)]. The simulations are based on the C_6_H_6_ potential energy surface (PES) and kinetic mechanism proposed by Miller and Klippenstein (*31*); the simplified PES depicting the most important channels of the propargyl radical self-reaction is illustrated in Fig. 5, but the full C_6_H_6_ PES including all three possible initial collision complexes was included in our kinetic calculations. Briefly, two propargyl radicals can barrierlessly recombine tail-to-tail (CH_2_ to CH_2_), head-to-head (CH to CH), or head-to-tail (CH to CH_2_) forming initial complexes **(2)**, **(5)**, and **(6)**, respectively. Intermediate **(2)** (1,5-hexadiyne) can isomerize to **(5)** (1,2,4,5-hexatetraene) via a six-member ring transition state; **(5)** then undergoes a five-member ring closure accompanied with a 1,2-hydrogen shift in the ring producing fulvene **(3)**. Alternatively, **(6**) (1,2-hexadiene-5-yne) can rearrange to **(4)** (2-ethynyl-1,3-butadiene) via a five-member ring transition state; **(4)** may further undergo a hydrogen shift and five-member ring closure to fulvene **(3)**. Fulvene can isomerize to benzene **(1)** via multiple pathways discussed by Miller and Klippenstein (*31*), but the most favorable pathway involves the formal insertion of the methylene group into the five-member ring leading to ring expansion; this pathway shown in Fig. 5 proceeds via intermediate **(7)**. Benzene can lose a hydrogen atom to form the phenyl radical **(9)** without an exit barrier. **(7)** can undergo molecular hydrogen elimination to *ortho* benzyne **(8)**; however, according to the Rice-Ramsperger-Kassel-Marcus (RRKM)-Master Equation (ME) calculations by Miller and Klippenstein (*31*), this channel is negligible under any conditions. Considering the complexity of this surface, which in their calculations included 16 potential wells and 21 saddle points resulting in 104 rate constants, Miller and Klippenstein proposed a simplified, “lumped” model for the propargyl self-recombination applicable for combustion conditions. According to their RRKM-ME results, at combustion temperatures (1500 K), the initial collision complexes **(2)**, **(5)**, and **(6)** react on time scales of a few microseconds and are stabilized as benzene **(1)**, fulvene **(3)**, and 2-ethynyl-1,3-butadiene **(4)** or dissociate to phenyl **(9)** plus atomic hydrogen. This conclusion was confirmed by our present calculations. For instance, at 1250 K and 30 torr, which are representative conditions inside the heated zone of the reactor, the calculated relative yields of the collision complexes **(2)**—1,5-hexadiyne, **(5)**—1,2,3,4-hexatetraene, and **(6)**—1,2-hexadiene-5-yne do not exceed 1.3%, whereas benzene, fulvene, and 2-ethynyl-1,3-butadiene are the predominant C_6_H_6_ isomers produced. In Miller and Klippenstein’s lumped kinetic model (*31*), groups of intermediates were merged together, with the most stable representatives of these groups being benzene **(1)**, fulvene **(3)**, and 2-ethynyl-1,3-butadiene **(4)**. Since 1,5-hexadiyne was observed in the present experiment, in our model, we separated this isomer from the other groups and considered its reactions distinctly. Alternatively, 1,2,3,4-hexatetraene and 1,2-hexadiene-5-yne were not detected and hence they were not considered separately. In the lumped model, 1,2,3,4-hexatetraene belongs to the 2-ethynyl-1,3-butadiene group, whereas 1,2-hexadiene-5-yne is equally split between the fulvene and benzene groups. Using this simplified kinetic model, we followed concentrations of the four C_6_H_6_ isomers **(1)**, **(3)**, **(4)**, and **(2)** in the reactor and the CFD—kinetic simulations were able to closely reproduce the experimental branching ratios for dilute propargyl radicals using source conditions of 0.7 and 1.0% precursor concentration. It should be noted that the results are not sensitive to the reactions (R11) to (R15) (table S4), which may be excluded for the kinetic mechanism as they do not play any substantial role in the product distribution. The RRKM-ME calculations by Miller and Klippenstein revealed that the dependence of the product branching ratios with temperature and pressure is very complex. The present experimental results show a preference for benzene formation at the lowest pressure (conditions 1; table S2) apparently when the entrance intermediates 1,5-hexadiyne and 2-ethynyl-1,3-butadiene are less prompt to collisional stabilization and the reaction proceeds further to fulvene **(3)** and then to benzene **(1)** with the computed branching ratios of methylacetylene, allene, distinct C_6_H_6_ isomers, and *o*-benzyne being close to the experimental values (Fig. 4 and table S3). To reproduce a relatively high yield of 1,5-hexadiyne **(2)** deduced from the experimental data, the nominal RRKM-ME calculated rate constants for the formation/consumption of **(2)** have to be adjusted significantly in favor of the formation processes. However, the experimentally derived branching ratios strongly depend on the absolute ionization cross sections of different C_6_H_6_ isomers, not all of which have been firmly established; for instance, the photoionization cross sections of fulvene and 2-ethynyl-1,3-butadiene are estimated or theoretically calculated. Therefore, the need for the adjustment of these rate constants may be reconsidered in the future when more precise photoionization cross sections for all C_6_H_6_ isomers become available.

### Implications and outlook

The first in situ detection of benzene [C_6_H_6_ **(1)**] along with three structural isomers [1,5-hexadiyne **(2)**, fulvene **(3)**, and 2-ethynyl-1,3-butadiene **(4)**] and *o*-benzyne **(8)** has far-reaching implications to the chemistry of aromatic systems in extreme environments spanning high-temperature combustion flames and carbon-rich circumstellar envelopes along with low-temperature, hydrocarbon-rich atmospheres of planets and their moons such as Titan and molecular clouds. First, cold molecular clouds, such as TMC-1 and OMC-1, are characterized by low temperatures of typically 10 K and number densities from 10^4^ to 10^6^ cm^−3^. Under these conditions, only bimolecular reactions without an entrance barrier proceed, and three-body collisions involving the stabilization of reaction intermediate(s) are absent (*42*). Consequently, in molecular clouds, once two propargyl radicals react without an entrance barrier, the initial collision complexes **(2)**, **(5)**, and/or **(6)** eventually isomerize to benzene and undergo unimolecular decomposition to the phenyl radical plus atomic hydrogen (Fig. 5). The reaction product of the propargyl radical self-reaction in cold molecular clouds—the phenyl radical (*52*)—together with more complex aryl radicals like 1-/2-naphthyl (*40*) have emerged as key reactants upon reaction with vinylacetylene (C_4_H_4_) leading via barrier reactions through ring annulation to rapid molecular mass growth processes to PAHs such as naphthalene, anthracene, and phenanthrene (*53*). Neither benzene **(1)** nor any of its isomers **(2)** to **(7)** can be formed via the propargyl-propargyl radical reaction in interstellar clouds. Recent crossed beam studies complemented with electronic structure calculations and astrochemical modeling revealed that in molecular clouds, benzene represents the product of the bimolecular reaction of the ethynyl radical (C_2_H) with 1,3-butadiene (C_4_H_6_) (*54*). Upon the barrierless elementary reaction of benzene with the cyano radical (CN), cyanobenzene (C_6_H_5_CN) can be prepared easily in molecular clouds (*55*) as verified through its recent astronomical detection toward TMC-1 (*56*). Second, hydrocarbon-rich atmospheres of planets and their moons such as of Titan are more complex. Similar to molecular clouds, the low atmospheric temperatures of, e.g., 70 to 180 K for Titan, support barrierless reactions. However, the denser atmospheric environments also support three-body collisions and hence the stabilization of, e.g., C_6_H_6_ reaction intermediates. The RRKM-ME calculations by Miller and Klippenstein suggested that at 100 torr—the relevant atmospheric range in Titan’s atmosphere corresponding to altitudes from 30 to 50 km—few of the collision complexes eventually undergo unimolecular decomposition to the phenyl radical [C_6_H_5_^•^ **(9)**] plus atomic hydrogen, whereas almost all can be stabilized by a third-body collision to distinct C_6_H_6_ isomers. Current atmospheric models of Titan properly implement barrierless propargyl-propargyl radical reactions; however, these models neither address appropriately the temperature- and pressure-dependent branching ratios of bimolecular versus three-body reactions nor account for discrete C_6_H_6_ isomers such as **(1)**, **(2)**, **(3)**, and **(4)** as detected in the present work, but operate under the hypothesis that solely benzene **(1)** is formed (*57*, *58*). Nevertheless, even in Titan’s atmosphere, benzene **(1)** can be easily photolyzed to the phenyl radical plus atomic hydrogen, thus eventually providing a fundamental building block to more complex PAHs such as naphthalene and beyond. Last, high-temperature combustion processes and carbon-rich circumstellar environments portray a similar complexity as hydrocarbon-rich atmospheres of planets and their moons (*59*). Miller and Klippenstein explored rate constants for the formation of C_6_H_6_ isomers via third-body collisions and bimolecular reactions leading to phenyl (C_6_H_5_^•^) plus atomic hydrogen over a broad range of temperatures (300 to 2200 K) and pressures (1 to 10^4^ torr). Similar to hydrocarbon-rich atmospheres, the product branching ratios strongly depend on the reaction conditions. For example, at 1000 K and 30 torr, benzene **(1)**, fulvene **(3)**, and phenyl **(9)** were predicted to be formed at fractions of 10, 18, and 5%, respectively. As pressures increase, the branching ratios of, e.g., benzene **(1)** and fulvene **(3)** drop, whereas one of the initial collision complexes—1,5-hexadiyne **(2)**—increases. Conversely, a temperature increase favors the formation of, e.g., phenyl **(9)** plus atomic hydrogen, benzene **(1)**, and fulvene **(3)**, which are predicted to be the main products of the propargyl radical self-reaction at 1500 K and 30 torr.

To conclude, the very first in situ identification of benzene [C_6_H_6_ **(1)**] together with three structural isomers [1,5-hexadiyne **(2)**, fulvene **(3)**, and 2-ethynyl-1,3-butadiene **(4)**] has fundamental implications to the chemistry and specifically to the formation pathways of benzene as the simplest building block of PAHs in interstellar, solar system, and combustion environments. Isomer-selective combustion and atmospheric models of, e.g., Titan, are desirable and imperative to correctly replicate the observations of aromatic species and of benzene in particular in combustion systems and the interpretation of data obtained from the Cassini-Huygens mission to Titan. Furthermore, an isomer-selective reaction pathway to an interstellar and circumstellar hydrocarbon chemistry has to be accounted for to properly reproduce astronomical observations and to gain a better understanding of the (low temperature) hydrocarbon chemistry in our universe.

## MATERIALS AND METHODS

The experiments were carried out at the Advanced Light Source at the Chemical Dynamics Beamline (9.0.2.) using a chemical reactor (*60*). Briefly, the high-temperature chemical reactor was a resistively heated SiC tube of 20-mm length and 1-mm inner diameter. In each experiment, the precursor propargyl bromide (C_3_H_3_Br) (Sigma-Aldrich; > 98%) was kept in a bubbler at 199 ± 1 or 233 ± 1 K and seeded in pure helium at inlet pressures of 200 or 300 torr. Each gas mixture was then expanded into a resistively heated SiC tube (pyrolytic reactor) with the temperatures monitored by a Type C thermocouple. Thus, four sets of experiments were carried out with the experimental conditions compiled in table S2. The gas mixture was introduced into the SiC tube at well-defined temperatures monitored by a Type C thermocouple. After exiting the reactor, the molecular beam passed a skimmer and entered a main chamber, which housed the Wiley-McLaren Re-TOF-MS. The products were photoionized in the extraction region of the reflectron time-of-flight spectrometer and detected with a microchannel plate detector. VUV single photon ionization represents essentially a fragment-free ionization technique (*61*). Here, mass spectra were taken in 0.10 eV intervals from 8.00 to 10.50 eV for the experiments with bubbler held at 199 K at a pressure of 200 torr. For other experiments, mass spectra were taken in 0.05 eV intervals within the same photon energy range. Because of the dilute precursor concentration, the signal in these experiments is very weak, and extended data accumulation times of up to 10 min per step have to be accounted for. The PIE curves, which report the intensity of a single *m/z* versus the photon energy, were extracted by integrating the signal collected at a specific *m/z* selected for the species of interest over the range of photon energies and normalized to the incident photon flux. Since the ion count normalized by the photon fluxes holds a direct proportional relationship with the concentration, the photoionization cross section, mass discrimination, and the ion counts measured in the experiment [*S*_i_(*T*, *E*) ∝ *X*_i_(*T*) ∙ σ_i_(*E*) ∙ *D*_i_] (*61*), the branching ratios between the concentrations of individual products can be calculated via ($\frac{X_{i}(T)}{X_{j}(T)}=\frac{S_{i}(T,E)}{S_{j}(T,E)}∙\frac{\sigma_{j}(E)}{\sigma_{i}(E)}∙\frac{D_{j}}{D_{i}}$). The mass discrimination factors were taken from (*62*).

All rate constants within the C_3_H_3_^•^ + C_3_H_3_^•^ → C_6_H_6_ → C_6_H_5_^•^ + H system were recomputed using the same RRKM-ME approach and the energetics and molecular parameters from Miller and Klippenstein’s work (*31*), whereas the high-pressure limit rate constants for barrierless C_3_H_3_^•^ + C_3_H_3_^•^ and C_6_H_5_^•^ + H recombinations were adjusted (using phase space theory) to match the best available theoretical values obtained from variable reaction coordinate transition state theory (VRC-TST) calculations (*63*, *64*). After the lumping (described in the CFD and kinetic simulations section), the reactions in the C_3_H_3_^•^ + C_3_H_3_^•^ → C_6_H_6_ → C_6_H_5_^•^ + H system were included in the present mechanism as reactions R3 to R7 and R17 (table S4). The overall kinetic mechanism also incorporated the decomposition of the bromopropargyl precursor (reaction R1), the recombination of propargyl with H atoms producing allene and propyne (R2), H atom abstraction from benzene by H (C_6_H_6_ + H → C_6_H_5_^•^ + H_2_, R8), H-assisted isomerization of fulvene to benzene (R9), decomposition of phenyl radical to C_6_H_4_ + H (R10), decomposition of bromobenzene (R11), H abstractions from phenyl by Br and H atoms (R12 and R13, respectively), recombination of two phenyl radicals to biphenyl (R14) and their disproportionation to benzene + benzyne (R15), and recombination of phenyl and propargyl radicals (R16). Rate constants for most reactions were taken from the literature as specified in table S4, whereas for C_3_H_3_Br ⇆ C_3_H_3_^•^ + Br, the rate constant was computed using the VRC-TST approach (*65*, *66*) before adjusting the C-Br bond strength to achieve the full decomposition of the bromopropargyl precursor observed under the experimental conditions. The rate constants were adjusted, as described and shown in table S4 to reproduce the measured branching ratios of the C_3_H_4_ and C_6_H*_x_* products. The change in the mole fractions of the components of interest is shown in Fig. 4. The efficient pyrolysis of C_3_H_3_Br and formation of C_6_H_6_ isomers start at the distance of nearly 1.8 cm downstream from the gas inlet into the SiC tube. At this point, the gas temperature reaches 1200 K and the C_3_H_3_Br decomposition rate approaches 2 × 10^4^ s^−1^. A further increase of the gas temperature results in an increase of the rate of the C_3_H_3_Br pyrolysis. The gas flight time between *x* = 1.8 cm and the tube outlet is on the order of 100 μs. This time is sufficient for efficient C_3_H_3_Br decomposition. The rates of production of C_6_H_6_ isomers are proportional to the rate constants of the appropriate subchannels of reaction R4. The production of C_6_H_4_ in the fast reaction R10 in the interval [2, 2.7] cm follows the production of C_6_H_5_^•^ in reaction R3. Downstream from 2.7 cm, the temperature and the C_3_H_3_ number density decrease, the rate of C_6_H_4_ production in sequences of reactions R3 and forward R10 falls down, while the recombination rate C_6_H_4_ + H in the reverse reaction R10 increases. This results in a decrease of the C_6_H_4_ fraction in the interval [2.7, 3.8] cm. The values of *C*_i_ at the outlet of the SiC tube were used to calculate relative branching ratios of the C_6_H_6_ isomers and C_6_H_4_

$$f_{i}=\frac{C_{i}}{\sum_{i=1}^{5}C_{i}}$$ (4)

These fractions for the experiments are presented in table S3.

## SUPPLEMENTARY MATERIALS

Supplementary material for this article is available at http://advances.sciencemag.org/cgi/content/full/7/21/eabf0360/DC1
